# Microbiological eradication of XDR *Acinetobacter baumannii* intra-abdominal infection using sulbactam-durobactam: clinical and microbiological outcomes amidst irreversible host comorbidities

**DOI:** 10.3389/fmed.2026.1837081

**Published:** 2026-05-15

**Authors:** Lei Yu, Jieli He, Waiming Chan, Shan Zou, Qingshan Zhou, Jun Jin

**Affiliations:** 1 Department of Intensive Care Unit, The University of Hong Kong - Shenzhen Hospital, Shenzhen, China; 2 Department of Pharmacy, The University of Hong Kong - Shenzhen Hospital, Shenzhen, China; 3 Critical Care Medicine Unit, LKS Faculty of Medicine, The University of Hong Kong, Hong Kong, Hong Kong SAR, China

**Keywords:** complicated intra-abdominal infection, CRAB, critical illness polyneuropathy, Kennedy’s disease, sulbactam-durlobactam

## Abstract

**Background:**

The management of extensively drug-resistant (XDR) *Acinetobacter baumannii* (CRAB) in deep-seated infections remains a critical challenge, particularly when compounded by severe host comorbidities.

**Case presentation:**

We report the case of a 63-year-old male with Stage IV rectal squamous cell carcinoma and Kennedy’s disease (spinal and bulbar muscular atrophy) who developed concurrent pulmonary and intra-abdominal infections with CRAB and carbapenem-resistant *Pseudomonas aeruginosa* (CRPA) following emergent colorectal surgery. After the failure of an initial salvage regimen comprising polymyxin B, minocycline, and ampicillin-sulbactam, therapy was escalated to sulbactam-durlobactam combined with imipenem-cilastatin.

**Results:**

Microbiological cure was achieved within 14 days, confirmed by the sterilization of both bronchoalveolar lavage fluid and peritoneal drainage cultures. Despite successful source control and infection eradication, the patient could not be weaned from mechanical ventilation. The convergence of critical illness polyneuropathy/myopathy (CIP/CIM), pre-existing neuromuscular degeneration, and malignant cachexia resulted in fatal multiple organ dysfunction syndrome.

**Conclusion:**

This case suggests the potential utility of sulbactam-durlobactam in combination with a carbapenem for complicated intra-abdominal infection (cIAI) caused by CRAB, highlighting a successful microbiological outcome. However, it also underscores that microbiological eradication does not invariably translate to clinical survival when overwhelming host factors and irreversible comorbidities dictate the ultimate prognosis.

## Introduction

Carbapenem-resistant *Acinetobacter baumannii* (CRAB) and Carbapenem-resistant *Pseudomonas aeruginosa* (CRPA) are designated by the World Health Organization (WHO) as critical-priority pathogens (1). According to the comprehensive 2019 Global Burden of Disease (GRAM) report, these organisms are among the leading pathogens driving antimicrobial resistance (AMR)-related mortality worldwide (2). They are frequently implicated in ventilator-associated pneumonia (VAP), bloodstream infections and cIAI (3). Recent global prospective cohort data demonstrate that CRAB infections carry a substantial 30-day all-cause mortality rate averaging 24% globally, which can surge to nearly 50% in certain highly endemic regions, with respiratory and bloodstream infections being the most prevalent (4). Patients undergoing major abdominal surgery for malignancy are particularly vulnerable to these multidrug-resistant pathogens due to immunosuppression, prolonged hospitalization, and the presence of invasive devices (5).

Traditional salvage therapies, such as polymyxins and tigecycline, are often limited by dose-dependent nephrotoxicity, neurotoxicity, and suboptimal pharmacokinetic penetration into the peritoneal cavity (6, 7). Sulbactam-durlobactam (SUL-DUR), a novel β-lactam/β-lactamase inhibitor combination, demonstrated non-inferiority to colistin for the treatment of CRAB pneumonia in the ATTACK trial (8). However, clinical data supporting its efficacy in managing deep-seated, extra-pulmonary infections specifically within the intra-abdominal compartment remains scarce.

Furthermore, the eradication of pathogens does not invariably translate to patient survival. Patients with pre-existing neuromuscular disorders, such as Kennedy’s disease, face a “double hit” when critical illness CIP/CIM complicates recovery, rendering ventilator weaning exceptionally difficult (9, 10). Herein, we present a case of a patient with metastatic rectal carcinoma and Kennedy’s disease who achieved microbiological cure of extensively drug-resistant (XDR) CRAB peritonitis and pneumonia using SUL-DUR, illustrating both the potential of novel therapeutics and the stark limitations imposed by host physiological reserve.

## Case presentation

### Initial presentation and surgical course

A 63-year-old male presented to a local emergency department on 8 October 2025 (Day -40), exhibiting acute abdominal pain, distension, and altered mental status. His medical history was notable for coronary artery disease, hypertension, and genetically confirmed Kennedy’s disease (diagnosed in 2002). The latter had resulted in progressive proximal muscle atrophy and bulbar weakness, causing chronic respiratory impairment. Abdominal computed tomography (CT) revealed pneumoperitoneum indicative of visceral perforation. An emergent exploratory laparotomy was performed, involving cecostomy, left hemicolectomy, and appendectomy. Histopathology confirmed Stage IV poorly differentiated rectal squamous cell carcinoma with extensive lymphovascular invasion. Although less common than adenocarcinoma, primary SCC of the rectum is a recognized entity often associated with chronic inflammation or HPV infection; the patient had no prior anal canal primary.

### Postoperative complications at referring hospital

The postoperative course was complicated by septic shock, respiratory failure necessitating mechanical ventilation, and a left upper lobe pulmonary embolism (confirmed by CT pulmonary angiography on October 17). Over a 40-day period, the patient suffered recurrent nosocomial infections involving multidrug-resistant organisms, including *Acinetobacter baumannii* and *Staphylococcus aureus*. Despite aggressive treatment with meropenem, tigecycline, and polymyxin B, weaning from mechanical ventilation failed due to the severity of the pulmonary infection and baseline neuromuscular weakness.

### ICU admission and microbiological identification

On 17 November 2025 (Day 1), the patient was transferred to our intensive care unit (ICU). He exhibited only low-grade fever. Bronchoalveolar lavage fluid (BALF) obtained on 21 November (Day 5) grew CRAB but was initially regarded as colonization and not treated. The blood cultures and blood targeted next-generation sequencing (t-NGS) remained consistently negative throughout the entire hospitalization.

Microbiological cultures of BALF and ascitic fluid were processed according to CLSI M100 guidelines. Bacterial identification was performed using MALDI-TOF MS (Bruker MALDI Biotyper, Germany). Antimicrobial susceptibility testing was conducted using broth microdilution or Kirby-Bauer disk diffusion method following CLSI standards (Table 1). For comprehensive resistance gene profiling, we employed our institutional t-NGS platform (PTseq Plus, BGI Genomics). The t-NGS analysis of the BALF sample revealed that the *Acinetobacter baumannii* harbored the blaOXA-23, blaOXA-51, blaTEM-1, AmpC, APH(3’)-VIa, and ANT resistance genes, whereas the *Pseudomonas aeruginosa* carried the AmpC and APH resistance genes. Due to the urgent clinical context clonal relatedness analysis was not performed. While the identical species identification suggests a possible clonal relationship between *Acinetobacter baumannii* isolates from different anatomical sites, this cannot be definitively confirmed without molecular typing.

**TABLE 1:** Antimicrobial susceptibility profile of the isolates.

Variables/isolates	CRAB-BAL MIC (μ g/ml)	CRAB-ascites MIC (μ g/ml)	CRPA-ascites MIC (μ g/ml)
Colistin	<1 (I)	<1 (I)	2 (I)
Cefoperazone-sulbactam	>32/8 (R)	>32/8 (R)	>64 (R)
Ceftazidime	>32 (R)	>32(R)	32 (R)
Piperacillin-tazobactam	>64/4 (R)	>64/4 (R)	>128 (R)
Ciprofloxacin	>4 (R)	>4 (R)	<0.25 (S)
Levofloxacin	>8 (R)	>8 (R)	1 (S)
Tobramycin	>8 (R)	>8 (R)	<1 (S)
Amikacin	>32 (R)	>32(R)	<2 (S)
Imipenem	>8 (R)	>8 (R)	>16 (R)
Meropenem	>8 (R)	>8 (R)	>16 (R)
Ticarcillin-clavulanate	-	-	>128 (R)
Cefepime	>16 (R)	>16(R)	16 (I)
Ceftazidime-avibactam	-	-	26 mm (S)^a^
Ampicillin-sulbactam	>16/8 (R)	>16/8 (R)	-
Ceftriaxone	>32 (R)	>32(R)	-
Gentamicin	>8 (R)	>8 (R)	-
Minocycline	8 (I)	8 (I)	-
Trimethoprim-sulfamethoxazole	>4/76 (R)	>4/76 (R)	-
Tigecycline	4 (I)	4 (I)	-
CRAB	POS	POS	-

CRAB, carbapenem-resistant *Acinetobacter baumannii*; CRPA, carbapenem-resistant *Pseudomonas aeruginosa*; BAL, bronchoalveolar lavage; MIC, minimum inhibitory concentration; S, susceptible; I, intermediate; R, resistant. Susceptibility interpretations are based on CLSI M100 breakpoints. Unless otherwise noted, MICs were determined by broth microdilution. Results annotated with “a” indicate results obtained by Kirby-Bauer disk diffusion method. “-” indicates not tested or data unavailable.

### Therapeutic intervention and clinical outcome

Source control was achieved via percutaneous drainage catheters into the abdominal collections. Adequacy of drainage was monitored daily by bedside ultrasound, which confirmed the progressive reduction of fluid collections without new abscess formation, ensuring that satisfactory mechanical source control was maintained concurrently with antimicrobial therapy. An initial salvage regimen comprising polymyxin B, minocycline, and ampicillin-sulbactam was administered. Despite these measures, the patient remained febrile with persistently elevated procalcitonin levels, and follow-up cultures from both respiratory and abdominal sites remained positive.

On 26 November (Day 9), upon the initiation of targeted therapy, the patient’s APACHE II score was 27, indicating severe critical illness. He was mechanically ventilated via tracheostomy on Pressure Support Ventilation (PSV) mode (PS 8 cmH2O, PEEP 5 cmH2O, FiO2 30%). Baseline laboratory parameters showed compensated organ function (Creatinine 21 μmol/L, AST 63 U/L) but active systemic inflammation (IL-6 104.7 pg/mL) (Supplementary Table 1). While the intra-abdominal infection was considered the dominant source, the evolving respiratory signs indicated that the BALF CRAB isolate had transitioned from a colonizer to a pathogen contributing to multifocal infection. Consequently, subsequent antimicrobial therapy targeted both the abdominal and pulmonary compartments. The antimicrobial regimen was escalated to SUL-DUR (1/1 g IV q6h) combined with imipenem-cilastatin (1/1 g q6h). This combination was selected to replicate the regimen investigated in the ATTACK trial, in which all patients received concomitant imipenem-cilastatin, and to align with the 2024 IDSA guidance recommending SUL-DUR plus a carbapenem as the preferred treatment for severe CRAB infections. While the co-infecting CRPA isolate showed *in vitro* resistance to imipenem (MIC > 16 μg/mL), the primary intent of this carbapenem was to enhance anti-CRAB bactericidal activity, with only an ancillary expectation of partial anti-pseudomonal effect in the context of optimized source control.

Following the initiation of SUL-DUR therapy on 26 November. On 1 December (5 days after therapy initiation), cultures from both BALF and ascitic fluid simultaneously became negative for CRAB. Sequential surveillance cultures for CRAB from both sites remained consistently negative throughout the remainder of the treatment course until its completion on December 9. Despite the achievement of confirmed microbiological eradication—evidenced by the sterilization of BALF and ascitic fluid, alongside consistently negative blood cultures and blood NGS—the patient did not achieve full clinical recovery. By the end of SUL-DUR therapy on December 9, although the APACHE II score had slightly decreased from 27 to 24, markers of systemic stress persisted. IL-6 levels decreased markedly from 104.7 to 55.1 pg/mL, and creatinine increased mildly from 21 to 45 μmol/L (Supplementary Table 1). This divergence highlights a critical paradigm in critical care: while the primary infectious insult was successfully neutralized microbiologically, the patient ultimately deteriorated due to the irreversible progression of underlying host factors, including malignant cachexia and Kennedy’s disease.

**FIGURE 1:**
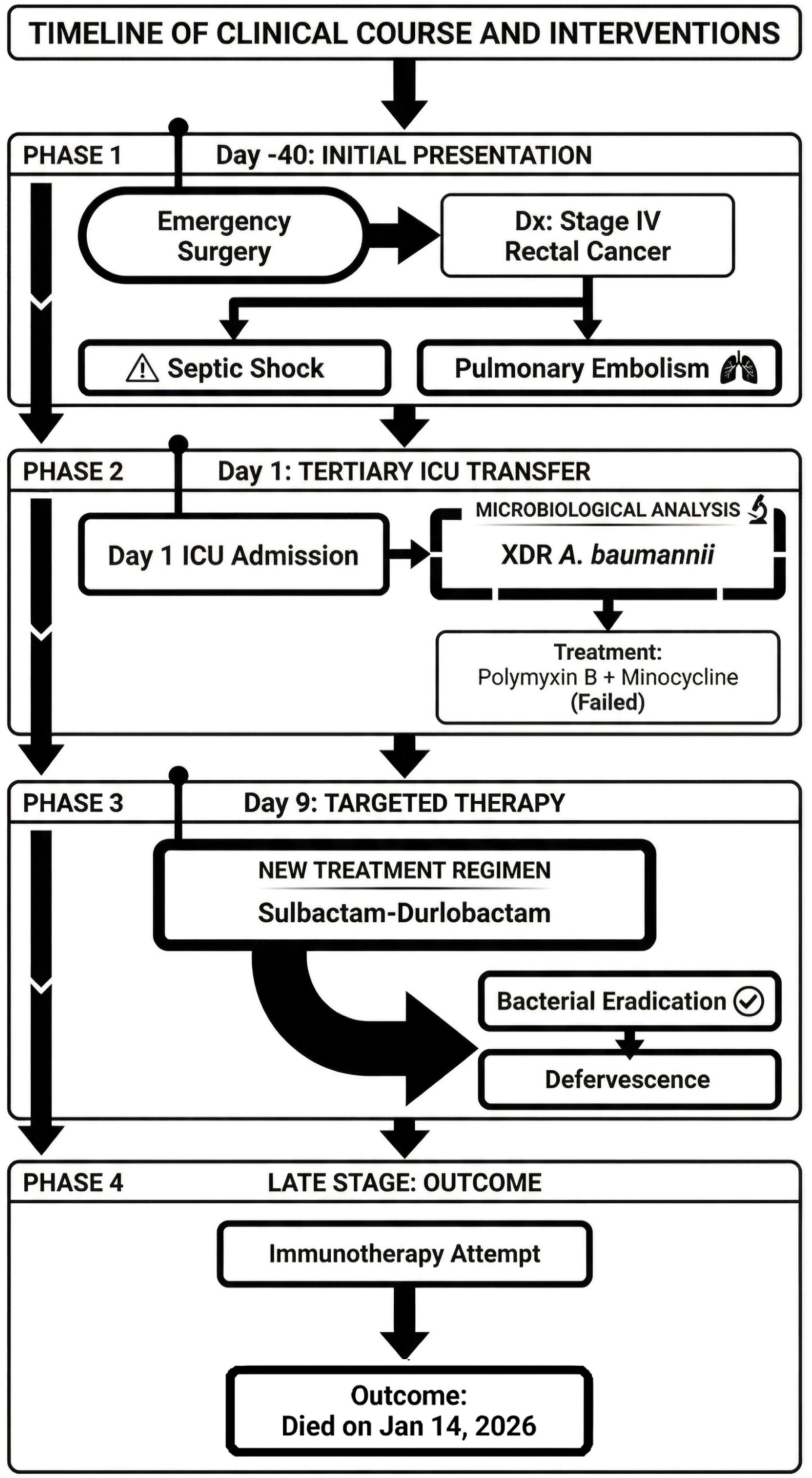
Timeline diagram of disease and treatment course.

Subsequently, immunotherapy with pembrolizumab was initiated for the underlying malignancy. However, the tumor exhibited rapid progression with widespread metastasis. The persistent failure to wean from mechanical ventilation was multifactorial. Despite being maintained on minimal support parameters (PSV mode, PS 8 cmH2O, PEEP 5 cmH2O) for an extended period, the patient could not sustain spontaneous breathing. While clinical signs strongly suggested the development of ICU-acquired weakness (ICUAW), likely encompassing critical illness CIP/CIM, this remained a clinical diagnosis. Formal electrophysiological testing could not be performed, and the clinical assessment of respiratory muscle weakness was profoundly confounded by the baseline motor neuron degeneration inherent to his Kennedy’s disease and malignant cachexia. He ultimately succumbed to multiple organ dysfunction syndrome (MODS) secondary to end-stage malignancy in mid-January 2026 (Figure 1).

## Discussion

This case highlights an association between the use of SUL-DUR and the successful microbiological eradication of concurrent CRAB pulmonary and intra-abdominal infections. While SUL-DUR was instrumental, the concurrent administration of imipenem-cilastatin and the continuous abdominal drainage (source control) were crucial adjunctive factors that undoubtedly contributed to the clearance of the local infection.

Carbapenem-resistant *Acinetobacter baumannii* and CRPA are formidable pathogens in intra-abdominal infections (IAIs), presenting significant treatment hurdles due to the encapsulation and poor vascular supply toperitoneal abscesses (2, 11). Polymyxins often achieve suboptimal concentrations in the peritoneal cavity and are limited by their narrow therapeutic indices (6). Mortality rates for XDR *Acinetobacter* peritonitis in the ICU frequently exceed 50% (12).

Durlobactam, a diazabicyclooctanone β-lactamase inhibitor, exhibits broad-spectrum activity against Ambler class A, C, and D enzymes, including OXA-23, OXA-24/40, and OXA-58 (13, 14). By protecting sulbactam from hydrolysis, durlobactam restores sulbactam’s intrinsic bactericidal activity against *Acinetobacter* via the inhibition of penicillin-binding proteins 1, 2, and 3 (7, 15). Pharmacokinetic data suggest plasma-to-peritoneal fluid ratios of 0.7–0.9 for sulbactam at steady state, supporting the feasibility of achieving bactericidal concentrations within the abdominal cavity (7, 16). This favorable tissue penetration provided the rationale for our off-label use of SUL-DUR in this refractory deep-seated infection (17).

The rapid clinical and microbiological response observed aligns with emerging real-world data regarding the efficacy of SUL-DUR in multi-site infections (8, 18). While durlobactam specifically targets *Acinetobacter*, its co-administration with imipenem-cilastatin was employed to provide synergistic coverage against the co-infecting CRPA, although intrinsic activity against *Pseudomonas* remains variable and requires susceptibility confirmation (19).

Despite achieving microbiological success, the patient’s mortality illustrates the phenomenon of “surviving sepsis but succumbing to frailty” (9). The convergence of CIP/CIM, Kennedy’s disease, advanced malignancy, and prolonged catabolism created a state of irreversible ventilator dependence (20). This case serves as a poignant reminder that while novel antimicrobials can effectively reverse septic shock and clear resistant pathogens, they cannot restore physiological reserves depleted by severe, chronic underlying disease.

This case report has several notable limitations. First, as a single observational case, the outcomes cannot be broadly generalized. Second, the concurrent use of imipenem-cilastatin and adequate source control act as significant confounding variables, precluding attribution of microbiological cure to SUL-DUR alone. Furthermore, the patient’s consistently negative blood cultures and blood mNGS indicate an absence of systemic bloodstream dissemination, meaning the efficacy of this regimen in CRAB bacteremia cannot be inferred from our case. Finally, we did not perform therapeutic drug monitoring (TDM) to measure specific SUL-DUR pharmacokinetic concentrations within the ascitic fluid, leaving its precise penetration profile in the abdominal compartment as an area requiring future formal pharmacological investigation.

## Data Availability

The original contributions presented in this study are included in this article/Supplementary material, further inquiries can be directed to the corresponding author.

## References

[1] Tacconelli E Carrara E Savoldi A Harbarth S Mendelson M Monnet D et al. Discovery, research, and development of new antibiotics: the WHO priority list of antibiotic-resistant bacteria and tuberculosis. *Lancet Infect Dis.* (2018) 18:318–27. 10.1016/S1473-3099(17)30753-3 29276051

[2] Antimicrobial Resistance Collaborators. Global burden of bacterial antimicrobial resistance in 2019: a systematic analysis. *Lancet.* (2022) 399:629–55. 10.1016/S0140-6736(21)02724-0 35065702 PMC8841637

[3] Marino A Augello E Bellanca C Cosentino F Stracquadanio S La Via L et al. Antibiotic therapy duration for multidrug-resistant gram-negative bacterial infections: an evidence-based review. *Int J Mol Sci.* (2025) 26:6905. 10.3390/ijms26146905 40725151 PMC12295917

[4] Wang M Ge L Chen L Komarow L Hanson B Reyes J et al. Clinical outcomes and bacterial characteristics of Carbapenem-resistant *Acinetobacter baumannii* among patients from different global regions. *Clin Infect Dis.* (2024) 78:248–58. 10.1093/cid/ciad556 37738153 PMC10874260

[5] Carmeli Y Armstrong J Laud P Newell P Stone G Wardman A et al. Ceftazidime-avibactam or best available therapy in patients with ceftazidime-resistant *Enterobacteriaceae* and *Pseudomonas aeruginosa* complicated urinary tract infections or complicated intra-abdominal infections (REPRISE): a randomised, pathogen-directed, phase 3 study. *Lancet Infect Dis.* (2016) 16:661–73. 10.1016/S1473-3099(16)30004-4 27107460

[6] Wong D Nielsen T Bonomo R Pantapalangkoor P Luna B Spellberg B. Clinical and pathophysiological overview of acinetobacter infections: a century of challenges. *Clin Microbiol Rev.* (2017) 30:409–47. 10.1128/CMR.00058-16 27974412 PMC5217799

[7] Alarcia-Lacalle A Canut-Blasco A Solinís M Isla A Rodríguez-Gascón A. Clinical efficacy, safety and pharmacokinetics of novel β-lactam/β-lactamase inhibitor combinations: a systematic review. *JAC Antimicrob Resist.* (2025) 7:dlaf096. 10.1093/jacamr/dlaf096 40583996 PMC12204647

[8] Kaye K Shorr A Wunderink R Du B Poirier G Rana K et al. Efficacy and safety of sulbactam-durlobactam versus colistin for the treatment of patients with serious infections caused by *Acinetobacter baumannii*-calcoaceticus complex: a multicentre, randomised, active-controlled, phase 3, non-inferiority clinical trial (ATTACK). *Lancet Infect Dis.* (2023) 23:1072–84. 10.1016/S1473-3099(23)00184-6 37182534

[9] Hiser S Casey K Nydahl P Hodgson C Needham D. Intensive care unit acquired weakness and physical rehabilitation in the ICU. *BMJ.* (2025) 388:e077292. 10.1136/bmj-2023-077292 39870417

[10] Shorter E Engman V Lanner J. Cancer-associated muscle weakness - from triggers to molecular mechanisms. *Mol Aspects Med.* (2024) 97:101260. 10.1016/j.mam.2024.101260 38457901

[11] Bonomo R Chow A Edwards M Humphries R Tamma P Abrahamian F et al. 2024 clinical practice guideline update by the infectious diseases society of America on Complicated intra-abdominal infections: risk assessment, diagnostic imaging, and microbiological evaluation in adults, children, and pregnant people. *Clin Infect Dis.* (2024) 79:S81–7. 10.1093/cid/ciae346 38965057

[12] Luo Q Chang M Lu P Guo Q Jiang X Xiao T et al. Genomic epidemiology and phylodynamics of *Acinetobacter baumannii* bloodstream isolates in China. *Nat Commun.* (2025) 16:3536. 10.1038/s41467-025-58772-9 40229304 PMC11997098

[13] Miller A Moussa S McLeod S. Characterization of *Acinetobacter baumannii*-calcoaceticus complex isolates and microbiological outcome for patients treated with sulbactam-durlobactam in a phase 3 trial (ATTACK). *Antimicrob Agents Chemother.* (2024) 68:e0169823. 10.1128/aac.01698-23 38567976 PMC11064521

[14] McLeod S Moussa S Hackel M Miller A. In vitro activity of sulbactam-durlobactam against *Acinetobacter baumannii*- calcoaceticus complex isolates collected globally in 2016 and 2017. *Antimicrob Agents Chemother.* (2020) 64:e02534-19. 10.1128/AAC.02534-19 31988095 PMC7179289

[15] Yahav D Giske C Grâmatniece A Abodakpi H Tam V Leibovici L. New β-Lactam-β-lactamase inhibitor combinations. *Clin Microbiol Rev.* (2020) 34:e00115-20. 10.1128/CMR.00115-20 33177185 PMC7667665

[16] Barnes M Kumar V Bethel C Moussa S O’Donnell J Rutter J et al. Targeting multidrug-resistant acinetobacter spp.: sulbactam and the diazabicyclooctenone β-Lactamase inhibitor ETX2514 as a novel therapeutic agent. *mBio*. (2019) 10:e00159-19. 10.1128/mBio.00159-19 30862744 PMC6414696

[17] Bartal C Rolston K Nesher L. Carbapenem-resistant *Acinetobacter baumannii*: colonization, infection and current treatment options. *Infect Dis Ther.* (2022) 11:683–94. 10.1007/s40121-022-00597-w 35175509 PMC8960525

[18] Iovleva A Fowler V Doi Y. Treatment approaches for carbapenem-resistant *Acinetobacter baumannii* infections. *Drugs.* (2025) 85:21–40. 10.1007/s40265-024-02104-6 39607595 PMC11950131

[19] Santerre Henriksen A Jeannot K Oliver A Perry J Pletz M Stefani S et al. In vitro activity of cefiderocol against European *Pseudomonas aeruginosa* and Acinetobacter spp., including isolates resistant to meropenem and recent β-lactam/β-lactamase inhibitor combinations. *Microbiol Spectr.* (2024) 12:e0383623. 10.1128/spectrum.03836-23 38483164 PMC10986614

[20] Vanhorebeek I Latronico N Van den Berghe G. ICU-acquired weakness. *Intensive Care Med.* (2020) 46:637–53. 10.1007/s00134-020-05944-4 32076765 PMC7224132

