# Supplementary material for: Microbiological eradication of XDR Acinetobacter baumannii intra-abdominal infection using sulbactam-durobactam: clinical and microbiological outcomes amidst irreversible host comorbidities
Source: Front Med (Lausanne). 2026 May 15;13:1837081. doi: 10.3389/fmed.2026.1837081 (PMC13218864; doi:10.3389/fmed.2026.1837081)
Supplement: Supplementary file 1 [file Table_1.DOCX]

Supplementary Table 1. Clinical and hematochemical parameters on day 1 and the last day of treatment with sulbactam-durlobactam

| Parameter | Day 1 (Nov 26)^a^ | Day 14 (Dec 9)^b^ | Unit |
| --- | --- | --- | --- |
| Clinical Severity Score |  |  |  |
| APACHE II | 27 | 24 |  |
| Arterial Blood Gas |  |  |  |
| pH | 7.475 | 7.471 |  |
| pCO2 | 5.03 | 4.35 | kPa |
| pO2 | 13.21 | 10.55 | kPa |
| sO2 | 97.9 | 95.2 | % |
| lactate | 3.35 | 3.92 | mmol/L |
| bicarbonate | 27.1 | 23.2 | mmol/L |
| Complete Blood Count |  |  |  |
| WBC | 24.66 | 26.02 | *10^9/L |
| hemoglobin | 76 | 69 | g/L |
| platelet count | 710 | 706 | *10^9/L |
| RBC | 2.78 | 2.49 | *10^12/L |
| organ function |  |  |  |
| total protein | 71 | 64 | g/L |
| albumin | 33 | 27 | g/L |
| ALT | 20 | 23 | U/L |
| AST | 63 | 76 | U/L |
| total bilirubin | 3.7 | <2.0 | umol/L |
| direct bilirubin | <2.9 | <1.0 | umol/L |
| creatinine | 21 | 45 | μmol/L |
| UREA | 4.42 | 15.34 | mmol/L |
| potassium | 4.4 | 4.5 | mmol/L |
| sodium | 134 | 140 | mmol/L |
| Inflammatory markers |  |  |  |
| procalcitonin | 0.74 | 0.44 | ng/ml |
| interleukin-6 | 104.7 | 55.1 | pg/ml |

^a^ Day 1 represents the baseline values on the day sulbactam-durlobactam therapy was initiated.
^B^ Day 14 represents the values on the day sulbactam-durlobactam therapy was completed.
Abbreviations: APACHE II:Acute Physiology and Chronic Health Evaluation II; 
pCO2: partial pressure of carbon dioxide;pO2:partial pressure of oxygen;sO2:oxygen saturation; WBC, white blood cell count; RBC, red blood cell count;ALT:alanine aminotransferase; AST: aspartate aminotransferase.
